# Dynamic interplay of developing internalising and externalising mental health from early childhood to mid-adolescence: Teasing apart trait, state, and cross-cohort effects

**DOI:** 10.1371/journal.pone.0306978

**Published:** 2024-07-10

**Authors:** Ioannis Katsantonis

**Affiliations:** Faculty of Education, Psychology, Education and Learning Studies Research Group, University of Cambridge, Cambridge, United Kingdom; University of Glasgow, UNITED KINGDOM OF GREAT BRITAIN AND NORTHERN IRELAND

## Abstract

The current study examined the within-child, between-child, and between-cohort effects in the longitudinal relations between and within the internalising and externalising mental health symptoms’ domains. Leveraging the data of 5998 children (ages 4, 6, 8, 10, 12, 14 years; 49% female) from the sequential Growing Up in Australia dual-cohort, multigroup longitudinal measurement invariance, and random-intercept cross-lagged panel models were deployed. Multigroup longitudinal measurement invariance revealed that the measurements of peer problems, emotional symptoms, and hyperactivity were strictly invariant, whereas conduct problems were partially strictly invariant across cohorts over time. The two cohorts did not display significant differences in the structural relations between internalising and externalising mental health symptoms, indicating the stability of the findings. In the internalising symptoms’ domain, moderate to strong reciprocal effects were found from middle childhood onwards. In the externalising symptoms’ domain, the results of reciprocal effects between conduct problems and hyperactivity were mainly not significant. Across domains, the reciprocal associations of emotional symptoms with hyperactivity and conduct problems were sporadic or non-existent. Peer problems were reciprocally associated with conduct problems and hyperactivity from middle childhood onwards. Overall, the findings clearly highlight the interdependence of developing internalising and externalising symptoms and reveal new insights about the early life-course development of internalising and externalising mental health symptoms.

## Introduction

Empirical studies examining the trends in the prevalence of children’s and adolescents’ mental health symptoms’ have noted an increase in emotional symptoms [1–4] and, specifically, in adolescents [2]. Additionally, epidemiological evidence suggests that one in eight children have a mental health disorder at any given time [5]. Evidence from the general population highlights that internalising symptoms (e.g., anxiety, depression) are more prevalent than externalising symptoms (e.g., hyperactivity, disruptive behaviour) [6]. The potential co-occurrence of internalising emotional and externalising behavioural symptoms [7,8] raises two important questions: Do internalising symptoms drive increases in externalising or vice versa? Or are these domains of psychopathology mutually reinforcing among themselves?

Despite the distinction between internalising and externalising symptoms, psychopathology studies suggest that these domains of mental health are interdependent [9–11]. In fact, studies suggest that there is a possibility of co-occurrence in internalising and externalising mental health symptoms [8,11,12]. Nevertheless, the literature records paradoxical results concerning the covariation between these domains of childhood psychopathology [12]. Given the above, the present study aims to contribute to ongoing debates about the long-term relations not only between but also within the domains of internalising and externalising mental health symptoms. Additionally, the study provides robust cross-cohort evidence by comparing two cohorts of Australian children born four years apart who participated in the Growing Up in Australia dual-cohort.

This article begins with a brief introduction to the theoretical perspectives underpinning the potential co-occurrence of symptoms. Afterwards, empirical literature on the co-occurrence is critically reviewed. Next, the trait-state approach to the study of co-occurrence between and within the internalising and externalising domains of psychopathology is discussed. The introductory section concludes with the description of the present study and the research questions. Methodological considerations are presented next, followed by the presentation of the results. The article culminates with a discussion of the findings and the implications of the study.

### Theoretical perspectives on the co-occurrence of internalising and externalising symptoms

The study of the developmental interdependencies between and within the domains of internalising and externalising mental health symptoms can be guided by theoretical research on the co-occurrence of childhood psychopathology [8]. Evidence coming from correlational [10,11,13] and behavioural genetics [14,15] empirical studies have shown a substantial correlation between the domains of internalising and externalising symptoms. However, co-occurrence does not only occur between domains but also within domains of internalising and externalising symptoms [7,16,17]. The within-domain co-occurrence is called homotypic, whereas the between-domain co-occurrence is called heterotypic and is more important from a substantive perspective [7].

Empirical explanations for this phenomenon of co-occurrence have been presented in the literature. One account, coming from the behavioural genetics literature, suggests that there is a genetic factor underpinning these associations that accounts for a large proportion of shared variance between externalising and internalising symptoms [12,15]. Another view suggests that internalising symptoms precede and predict subsequent externalising symptoms [7], whereas conduct problems in the form of antisocial behaviour can also give rise to internalising symptoms [7]. A dynamic mutualism view has been introduced, which suggests that symptoms of different mental health classifications can reinforce each other through direct causal links and interactions, instead of a common underlying cause [18]. This basically means that once weakly correlated symptoms can grow to be strongly associated over time as they influence each other across the development [18]. This dynamic mutualism viewpoint is usually examined through the ‘cascade effects’ model in psychopathology [18,19], which describes how psychopathology factors interact and transact between and within different developing levels (e.g., externalising and internalising) to modify the developmental course of a process [20]. So, a problem in one area can spread and cause issues in other areas, creating a chain reaction of difficulties.

Another explanation of the co-occurrence comes from studies using dimensional analyses through latent variable models that propose a general underlying psychopathology factor (usually called p-factor) that accounts for the co-occurrence within the domains of internalising [16] and externalising [21], as well as between the domains of internalising and externalising [18]. This general psychopathology factor is assumed to stem from higher-order dimensions of personality or temperament [7].

### Developmental relations between and within the internalising and externalising mental health domains

The study of the longitudinal developmental relations between facets of internalising and externalising mental health has attracted the attention of researchers. However, as will be shown, the results from the longitudinal studies are, to date, inconclusive and sometimes point in different directions.

Some early studies have noted no significant predictive effects (also known as cascading effects [20]) between internalising and externalising mental health in children or adolescents [22–24]. In contrast, other evidence has come to light indicating a unidirectional predictive effect [10,25,26]. Nevertheless, a few studies have also shown some small reciprocal effects. Such an example is the study by Wiggins et al. [27] that found a reciprocal association between internalising and externalising symptoms between ages 5 and 9. Similarly, another study reported significant reciprocal cascading effects between the two domains from age 3 to age 9 [13]. These findings suggest that a reciprocal cascading model might serve as a possible explanation of the long-term relations between children’s internalising and externalising mental health.

Beyond the studies on the between-domain relations, the evidence on relations within the domains of internalising and externalising mental health symptoms is also inconclusive and scarce. In the internalising domain, for example, a study found no longitudinal associations between emotional symptoms (i.e., depressed affect) and peer problems within children [28], whereas other studies have shown a longitudinal link between peer problems and emotional symptoms [29]. Some other evidence points toward a reciprocal longitudinal relation only between specific time points [30]. In the externalising domain, the evidence regarding the bidirectionality between conduct problems and hyperactivity is rather scarce, especially at the within-child level. A study from the behavioural genetics literature has reported a shared genetic underpinning to the relation between conduct problems and hyperactivity [31]. Longitudinal evidence has also highlighted the long-term predictive effect from hyperactivity indicators to conduct problems [32–34]. In short, some of the above evidence is rather short-term and does not cover all the developmental stages between early childhood and mid-adolescence.

The discrepancies in the conclusions reached across the empirical investigations suggest the need for replicating the findings through cross-validation. The importance of cross-validation of structural relations is a well-known property [35,36]; however, to the best extent of my knowledge, there are no previous studies on the cascading dynamics between and within internalising and externalising domains that have employed cross-validation cross-cohort approaches.

### A state-trait approach to the study of the dynamic interplay between and within internalising and externalising mental health domains

Having discussed the evidence coming from previous empirical studies on the longitudinal links between and within the domains of internalising and externalising symptoms, I now discuss the importance of trait-state approaches to the study of these longitudinal interdependencies. As mentioned in the literature, it is hard to think of psychological factors that are not simultaneously characterised by stability and change over the course of development [37]. Latent state-trait theory suggests that emotional responses, cognitions, and behavioural reactions in humans can be distinguished by stable traits, which describe stability over the course of development, and states, which reflect momentary deviations from the traits [38].

**Fig 1:**
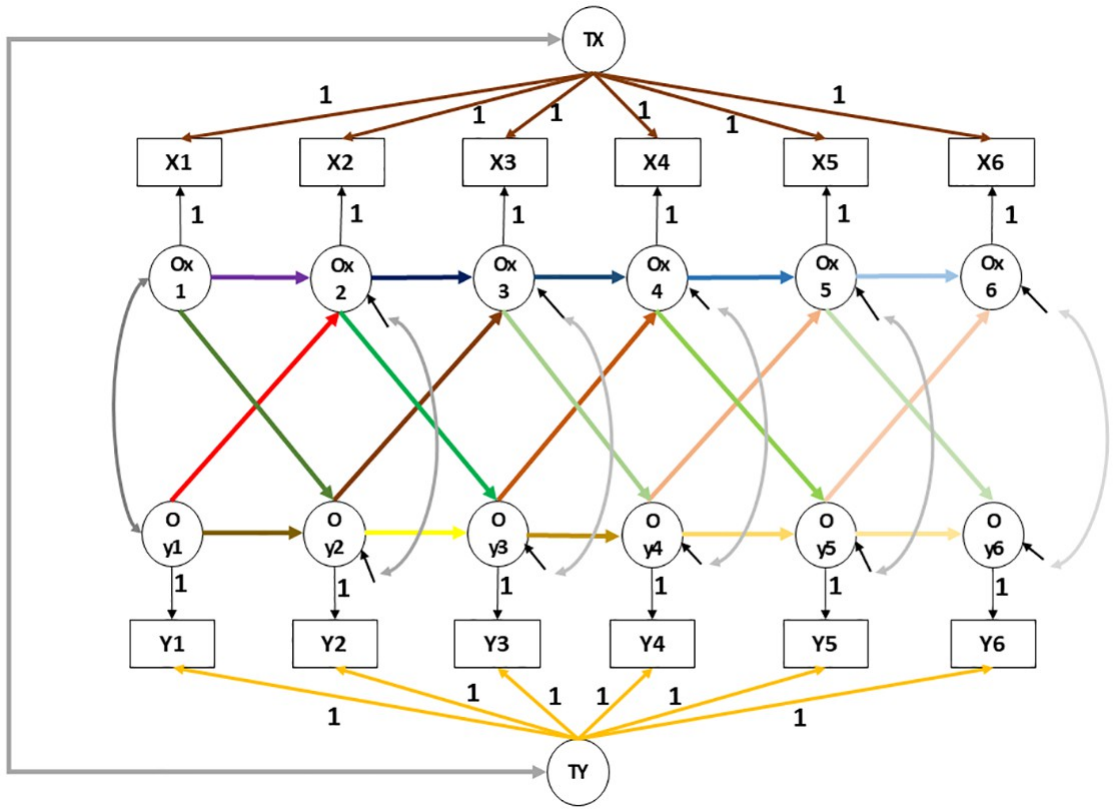
Path diagram of conceptual bivariate random-intercept cross-lagged panel model with six occasions from age 4 to age 14 years. X/Yt: Observed variable at time t; Oyt/ Oxt: Occasion-specific latent residual capturing momentary deviations from the trait; TY/TX: Stable trait latent variable capturing stable individual differences; Only labelled paths indicate equality of coefficients; All unlabelled paths are freely estimated.

The majority of the above studies on the interplay between internalising and externalising mental health symptoms have adopted a state-trait perspective [10,24,26], recognising that childhood internalising and externalising mental health symptoms are characterised by both stability and change over time. Hence, it is important to distinguish between inter-individual (also known as between-child) associations from intra-individual (also known as within-child) associations between and within the domains of internalising and externalising symptoms. To achieve this, the random-intercept cross-lagged panel model (RI-CLPM) [37,39] is deployed in the current study. The path configuration of a conceptual bivariate RI-CLPM is shown in Fig 1. In the RI-CLPM, the bi-factor reflects the stable trait-like aspects of symptoms, whereas the residual factors attached to the observable measures reflect the state-like aspects that are transient and subject to change [40]. The dynamic mutualism hypothesis of co-occurrence can be tested using the cross-lagged paths in the RI-CLPM, which indicate cascades between the different states.

In the framework of the state-trait approach [10,41], the general psychopathology underlying factor (p-factor) that emerges is conceptually similar to the correlation between the trait-like bi-factor structure of the RI-CLPM [37]. If the correlation between the different traits is very large and close to unity, then the traits will be indistinguishable, and the hypothesis of a common underlying trait factor over the course of development would hold [42]. Thus, the current study contributes to the ongoing discussion about the co-occurrence of internalising and externalising symptoms. This contribution stems from the examination of the co-occurrence at the within-child level and at the between-child /trait level over the course of the development from early childhood to mid-adolescence.

### The present study

The present study weaves together the theoretical perspectives on the co-occurrence between and within the internalising and externalising mental health domains [7,16,18] and the longitudinal state-trait perspective [38,41] to address two important questions. First, the study queries whether internalising symptoms drive developments in the externalising symptoms or vice versa. Second, the study explores whether these domains are mutually reinforcing as children develop over time. Reciprocal associations between and within the domains of internalising and externalising symptoms would lend support for the hypothesis of dynamic mutualism over time [18]. In contrast, a unidirectional flow of effects would indicate that one domain or component of psychopathology is the leading cause of the co-occurrence between and within the psychopathology domains [7].

The state-trait theoretical and modelling approach adds to the above viewpoints by disentangling the developmental dynamics of the symptoms within the children’s development from the general traits of psychopathology that remain stable over time [10]. Therefore, the current study provides insights into how children with greater than average symptoms in one domain might display greater than average increases in the other domain. In addition to the above, the study contributes by cross-validating the findings through a cross-cohort comparison, which can provide more conclusive evidence regarding the stability of the findings over time (2004–2014 vs. 2008–2018). Overall, the present study is guided by two research questions and the following hypotheses:

RQ1: Are the within-child longitudinal relations between emotional symptoms, peer problems, hyperactivity, and conduct problems comparable between cohorts?
RQ2: What is the longitudinal within-child association between emotional symptoms, peer problems, hyperactivity, and conduct problems between ages 4 and 14 years?

Provided that different longitudinal cohort studies reached competing conclusions, it is expected that cohort membership would moderate the longitudinal dynamic interplay between internalising and externalising symptoms (H1). The second research hypothesis assumes a positive relation between different facets of mental health symptoms (H2). Given the past findings, it is more likely that externalising symptoms will positively predict internalising symptoms (H3). However, some studies have also reported a positive reciprocal association between internalising and externalising mental health symptoms at specific ages; hence, a reciprocal association might be possible only between specific time points (H4).

## Materials and method

### Participants and data sets

The data come from the longitudinal accelerated Growing Up in Australia dual-cohort, also known as the Longitudinal Study of Australian Children (LSAC) [43]. Detailed information about the dual-cohort design and its methodologies can be found at https://growingupinaustralia.gov.au. Both the kindergarten (K) and the baby (B) cohorts’ data were utilised, leaving a total sample size of 5998 children. The baby cohort contributed 2722 (48.38% female) children, and the kindergarten cohort contributed 3276 (49.27% female) children. The baby cohort (infant) sampled children born between March 2003–February 2004, whereas the kindergarten cohort sampled children born between March 1999–February 2000 [43]. A two-stage stratified cluster sampling design was employed, whereby the clustering was with respect to the postal codes, and the stratification ensured the proportional selection from different geographical areas [43]. Children in both cohorts had approximately equal probabilities of selection [43]. About 60% of the parents were born in Australia, and more than 90% of the children were also born in Australia. The children in the current study were assessed biennially at ages 4, 6, 8, 10, 12, and 14. The baby cohort’s children were 4, 6, 8, 10, 12, and 14 years old between the calendar years 2008–2018, whereas the kindergarten cohort’s children were 4, 6, 8, 10, 12, and 14 years old between the calendar years 2004–2014.

### Measures

#### Strengths and difficulties questionnaire- emotional symptoms, conduct problems, hyperactivity, and peer problems

Internalising and externalising mental health was measured using the parent-reported four symptoms scales comprising the standardised Strengths and Difficulties Questionnaire (SDQ) [44,45]. The SDQ is a validated psychometric screening measure of children’s mental health difficulties [46], which has also been utilised in multiple longitudinal population studies in the UK [9,10] and Ireland [11]. The emotional symptoms scale comprises five items, such as “the [child] often complains of headaches.” The conduct problems scale is also made up of five items. A sample item of the conduct scale is “often fights with other children.” It should be noted that the conduct scale at age four years is slightly different because it contains two different items (see www.sdqinfo.org). The two different items at age 4 years are “[the child] is argumentative with adults” and “has been spiteful to others”. The hyperactivity scale also comprises five items. A sample hyperactivity item is “[the child] is restless, overactive”. Finally, the peer problems scale contains five items that measure problematic peer relations, such as “[the child] is rather solitary, tends to play alone”. All items were scored using 3-point scale ranging from 0 “not true” to 2 “certainly true”. A summed composite score index for each scale was created, with higher scores indicating greater difficulties in each domain. Emotional symptoms and peer problems belong to the domain of internalising symptoms, whereas conduct problems and hyperactivity are indicators of the externalising domain [47].

#### Covariates

*Child sex*. A single binary variable reflecting whether the children were female versus male.

*Mothers’ income at age four years*. A single ordinal variable ranging from 1 “less than $500 pw $25,999 or less per year” to 4 “$2,000 or more per week $104,000 or more per year”.

*Parental mental ill-health at age four years*. The Kessler 6 (K6) scale was utilised, and it was designed to screen adult community samples for mental illness [48]. A sample item is “how often have you felt restless or fidgety?”. Both mothers’ and fathers’ K6 scores were summed to form a composite of parental mental ill-health scores.

#### Ethics statement and consent

The study was conducted in accordance with the Helsinki Declaration of 1975, as revised in 2008. The Growing Up in Australia cohort received ethical approval from the Australian Institute of Family Studies Ethics Committee. This study is part of a larger project that has received ethics approval from the Faculty of Education, University of Cambridge, UK. Written consent was obtained. The author does not have access to any data that could be used to identify the participants. The data were accessed for research purposes on 8 October 2023.

#### Statistical analyses

**Fig 2:**
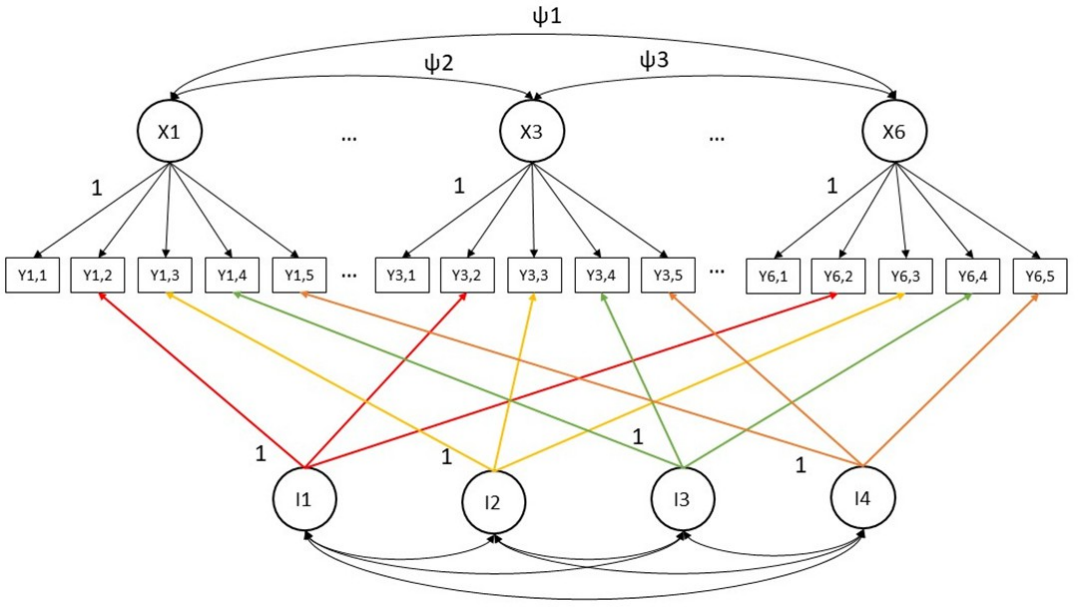
Reduced diagram of longitudinal multigroup measurement invariance configural model with k-1 indictor-specific method factors. X_t_ are common latent factors made up by repeated five items each; I_t_ are indicator-specific method factors capturing systematic method variance; ψ_t_ are covariances between the common latent factors.

The data from multiple waves were merged over time. In the first instance, multigroup longitudinal measurement invariance analyses (M_LMI) at the item level were performed to ensure the complete comparability of the measurements using the SDQ across the two LSAC cohorts. The single-group approach advocated for ordered-categorical data was adopted [49,50]. First, the multigroup longitudinal configural model was specified and tested, which signals that the latent factor structure of each SDQ scale is equivalent over time. In the configural model, the six latent factors were correlated, and the latent factor means were freely estimated from age six years onwards in the second group [49]. The unique variances of the first group were automatically fixed to 1 to achieve identification. Instead of specifying like-item residual correlations, which are problematic [51], correlated indicator-specific method factors (N_items_-1) were utilised to partial out shared method variance over time [51,52]. The indicator-specific method factors were held equal over groups, where possible. The path diagram of this model is shown in Fig 2. After establishing configural M_LMI, metric (i.e., loadings), scalar (i.e., thresholds), and strict (i.e., unique/residual variances) invariance levels were tested. An absolute difference (Δ) equal to/less than .01 and .015 in the CFI and RMSEA values, respectively, between two nested models indicates that invariance can be established [53].

Having tested the M_LMI, separate bivariate RI-CLPMs [37] were run. The advantage of the RI-CLPM over alternative cross-lagged panel models is that it clearly separates stable (‘trait-like’) individual differences from change within children (occasion-specific component), which is desirable in psychological research [40]. The autoregressive effects are also known as carry-over effects from a previous state to a subsequent state, whereas the cross-lagged effects are known as spillover effects from the previous state of one factor to the subsequent state of the other factor in the system [39].

To examine the effects of cohort membership/ time (2004–2014 vs. 2008–2018), multigroup RI-CLPMs were estimated with cohort membership as the grouping variable. Six multigroup bivariate RI-CLPMs were estimated between the composite indices of the four SDQ scales. Each multigroup RI-CLPM comprised a comparison between two nested models, namely an unconstrained model with freely estimated unstandardised autoregressive and cross-lagged coefficients and a constrained model with equality constraints on the unstandardised autoregressive and cross-lagged regressions [39]. A statistically significant Satorra-Bentler chi-square differences test would indicate that time/cohort membership had a moderating effect on the structural within-child relations [39]. To interpret the strength of the effect sizes, existing guidelines were followed [54]. Specifically, standardised within-child regression coefficients of .03 are small effects, coefficients of .07 are medium effects, and coefficients equal to/larger than .12 indicate large effect sizes [54]. Finally, as a robustness check, the three covariates were added into the multigroup RI-CLPMs as predictors of the random intercepts to examine whether the structural relations remained stable under the influence of contextual factors.

Measurement invariance analyses were run with the Weighted Least Squares Mean and Variance adjusted (WLSMV) estimator, which appropriately takes into account the ordered-categorical nature of the item-level data [55]. The multiple-indicator RI-CLPM with ordered-categorical data has neither been developed in the key RI-CLPM literature [37,39] nor has its properties been thoroughly studied through a large-scale simulation. A recent study has shown that it might be possible to estimate a simpler (ordered-categorical variables) RI-CLPM with the WLSMV, but this approach is prone to empirical under-identification, larger standard errors, and for some data sets, some parameters cannot be identified [56]. When multiple-indicator RI-CLPMs were estimated with WLSMV estimator, the models were prone to empirical under-identification for variances (Heywood cases), which are known properties of the WLSMV [57]. Furthermore, given the evidence of metric, strong, and strict invariance for emotional symptoms, peer problems, and hyperactivity over time and across groups and partial invariance for conduct problems, creation and comparison of composite scores is defensible [58]. The above reasons justify the estimation of the RI-CLPM with the robust maximum likelihood (MLR) estimator. To evaluate the fit of the structural and measurement models, the conventional cut-offs in approximate fit indices were adopted. Specifically, values close to .95 in the CFI and TLI indices, along with values below .06 and .08 in RMSEA and SRMR indices, respectively, indicate a very good fitting model to the data [59]. The available longitudinal sampling weights, the clustering, and the stratification design were taken into account in the inferential data analyses. Measurement invariance and RI-CLPMs were estimated in Mplus 8.7 [60], whereas preliminary data processing was done in Stata 17 [61]. The analytic code was uploaded to the Open Science Framework and can be viewed at https://osf.io/yjc27/?view_only=305264bcbd54464bb6e20a94c553b7f0.

## Results

### Descriptive statistics of key outcomes

Weighted descriptive statistics (means, standard deviations) were calculated for the kindergarten and baby cohorts and are presented in Table 1.

**Table 1:** Univariate descriptive statistics for the baby and kindergarten cohorts’ mental health indices.

Variable (time)	Mean (SD) Kindergarten cohort	Min-Max Kindergarten cohort	Mean (SD) Baby cohort	Min-Max Baby cohort
1. EM1	1.743 (1.695)	0–10	1.474 (1.538)	0–10
2. EM2	1.690 (1.747)	0–10	1.742 (1.794)	0–10
3. EM3	1.624 (1.807)	0–10	1.816 (1.883)	0–10
4. EM4	1.996 (1.994)	0–10	1.884 (2.024)	0–10
5. EM5	2.033 (1.986)	0–10	2.041 (2.057)	0–10
6. EM6	1.986 (2.008)	0–10	2.095 (2.118)	0–10
7. PP1	1.664 (1.539)	0–10	1.405 (1.458)	0–8
8. PP2	1.561 (1.577)	0–10	1.381 (1.530)	0–9
9. PP3	1.527 (1.653)	0–10	1.458 (1.583)	0–10
10. PP4	1.575 (1.750)	0–10	1.531 (1.704)	0–10
11. PP5	1.502 (1.670)	0–9	1.512 (1.712)	0–9
12. PP6	1.644 (1.715)	0–9	1.728 (1.820)	0–9
13. HYP1	3.520 (2.262)	0–10	3.391 (2.133)	0–10
14. HYP2	3.374 (2.284)	0–10	3.594 (2.362)	0–10
15. HYP3	3.252 (2.339)	0–10	3.538 (2.531)	0–10
16. HYP4	3.285 (2.381)	0–10	3.227 (2.449)	0–10
17. HYP5	3.063 (2.331)	0–10	3.116 (2.435)	0–10
18. HYP6	2.848 (2.291)	0–10	2.804 (2.336)	0–10
19. CON1	2.495 (2.009)	0–10	2.223 (1.783)	0–10
20. CON2	1.511 (1.504)	0–9	1.527 (1.465)	0–9
21. CON3	1.336 (1.482)	0–9	1.310 (1.520)	0–10
22. CON4	1.419 (1.532)	0–9	1.150 (1.483)	0–10
23. CON5	1.158 (1.503)	0–10	1.079 (1.535)	0–10
24. CON6	1.080 (1.530)	0–10	.992 (1.437)	0–9

*Note*: N baby cohort = 2722; N kindergarten cohort = 3276; EMt: Emotional symptoms at time t; PPt: Peer problems at time t; HYPt: Hyperactivity at time t; CONt: Conduct problems at time t; t = 1: Age 4 years; t = 2: Age 6 years; t = 3: Age 8 years; t = 4: Age 10 years: t = 5: Age 12 years; t = 6: Age 14 years; Min: Minimum observed value; Max: Maximum observed value.

### Preliminary psychometric analyses: Cross-cohort multigroup longitudinal measurement invariance

Multigroup longitudinal invariance analyses were first conducted to compare the dynamic interplay between the four mental health indices over time across cohorts and to ensure comparability of measurements [62]. The results of the multigroup longitudinal invariance analyses are presented in Table 2. As shown in Table 2, emotional symptoms, peer problems, and hyperactivity were strictly invariant across cohorts over time. Probably due to the two different item wordings of the conduct scale at age 4, conduct problems were only partially strictly invariant over time, with the problem of non-invariance being located at the items’ thresholds at age 4. The non-invariant thresholds at age four years signal that the first timepoint is likely to be an inefficient predictor in the cross-lagged modelling.

**Table 2:** Cross-cohort multigroup longitudinal invariance analyses’ results- comparisons between approximate fit indices.

Invariance level	CFI	|ΔCFI|	RMSEA	|ΔRMSEA|
	**Emotional symptoms**			
Configural	.960		.029	
Metric	.959	.001	.029	.000
Scalar	.954	.005	.030	.001
Strict	.952	.002	.030	.000
	**Peer problems**			
Configural	.977		.020	
Metric	.975	.002	.020	.000
Scalar	.974	.001	.020	.000
Strict	.970	.004	.021	.001
	**Hyperactivity**			
Configural	.987		.027	
Metric	.985	.001	.027	.000
Scalar	.984	.001	.027	.000
Strict	.985	.001	.026	.001
	**Conduct problems**			
Configural	.970		.023	
Partially Metric	.969	.001	.023	.000
Partially Scalar	.969	.000	.022	.001
Partially Strict	.969	.000	.022	.000

*Note*: N baby cohort: 2722; N kindergarten cohort: 3276; Due to different items in conduct problems at age 4, partially metric, scalar, and strict invariance were tested.

### Multigroup bivariate RI-CLPMs of mental health: Examining the moderating effect of time/cohort membership

The results of cross-cohort multigroup RI-CLPM model comparisons are presented in Table 3. As shown in Table 3, the multigroup models’ comparisons revealed no differences in the within-child structural relations across cohorts, given the statistically insignificant Satorra-Bentler tests. In fact, the constrained models exhibited a better fit in all models. This suggests that time had no moderating effect on the relations within and between the domains of internalising and externalising mental health and that any findings of relations hold across cohorts.

**Table 3:** Cross-cohort multigroup RI-CLPM models examining the moderating effect of time.

Model	SB Δχ^2^ (df)	CFI	TLI	RMSEA	SRMR
EM-PP (unconstrained)		.971	.955	.045	.043
EM-PP (constrained)	22.74 (20) *ns*	.971	.964	.040	.044
EM-HYP (unconstrained)		.966	.947	.056	.050
EM-HYP (constrained)	21.84 (20) *ns*	.967	.958	.049	.051
EM-CON (unconstrained)		.972	.957	.042	.045
EM-CON (constrained)	20.89 (20) *ns*	.973	.966	.037	.047
PP-HYP (unconstrained)		.969	.952	.054	.049
PP-HYP (constrained)	23.24 (20) *ns*	.970	.962	.048	.050
PP-CON (unconstrained)		.977	.965	.038	.041
PP-CON (constrained)	19.60 (20) *ns*	.978	.973	.033	.043
HYP-CON (unconstrained)		.977	.964	.047	.043
HYP-CON (constrained)	20.05 (20) *ns*	.977	.972	.041	.046

*Note*: ns: Not statistically significant; SB: Satorra-Bentler chi-square differences test; df: Degrees of freedom; EM: Emotional symptoms; PP: Peer problems; HYP: Hyperactivity; CON: Conduct problems.

### Within-domain and between-domains structural relations of internalising and externalising mental health

Having compared the within-child effects across cohorts, the standardised structural parameters were inspected next. The standardised regression coefficients were derived from the constrained multigroup models, which calculated separate but comparable covariance matrices and are presented in Table 4. I reiterate here that only the autoregressive and cross-lagged coefficients, respectively, were specified to be 1:1 equal across cohorts. Overall, rather modest within-child autoregressive carry-over effects were found between adjacent ages. This suggests that children’s mental health development fluctuated substantially over time; that is, those children who initially faced greater than usual difficulties did not necessarily persist in facing difficulties over time. In general, strong positive reciprocal relations were observed between emotional symptoms and peer problems from age eight years onwards. Reciprocal relations were also observed between emotional symptoms and hyperactivity only in the transitions between middle childhood (age 8) to early adolescence (age 10) and between early adolescence (age 12) to mid-adolescence (age 14). Between conduct problems and emotional symptoms, there were no significant reciprocal links. In contrast, consistent reciprocal relations were found between peer problems and hyperactivity from middle childhood (age 8 years) onwards. Higher than usual peer problems were moderately to strongly reciprocally associated with greater conduct problems from age eight years onwards. Within the externalising domain, the evidence was rather mixed, with few statistically significant reciprocal effects between hyperactivity and conduct problems, which were mostly concentrated in the adolescent years (between ages 12 and 14 years). Most effects from age 4 years to age 6 years were rather ambiguous, being insignificant or the opposite than expected sign. Finally, the trait correlations (i.e., correlations between random intercepts) consistently revealed that there was a robust positive association between children between and within the different components of internalising and externalising mental health.

**Table 4:** Standardised parameter estimates of the multigroup bivariate RI-CLPMs.

	β (S.E.)	β (S.E.)		β (S.E.)	β (S.E.)
	Autoregressive effects			Cross-lagged effects	
	B cohort	K cohort		B cohort	K cohort
**Model: EM-PP**					
EM1→EM2	.092 (.024)***	.107 (.028)***	EM1→PP2	.038 (.021)	.042 (.024)
EM2→EM3	.267 (.031)***	.259 (.029)***	EM2→PP3	.106 (.025)***	.099 (.024)***
EM3→EM4	.318 (.026)***	.320 (.025)***	EM3→PP4	.106 (.024)***	.100 (.023)***
EM4→EM5	.350 (.026)***	.353 (.024)***	EM4→PP5	.097 (.021)***	.097 (.021)***
EM5→EM6	.435 (.021)***	.440 (.020)***	EM5→PP6	.138 (.021)***	.147 (.022)***
PP1→PP2	.102 (.024)***	.100 (.024)***	PP1→EM2	.034 (.023)	.036 (.024)
PP2→PP3	.184 (.026)***	.184 (.027)***	PP2→EM3	.042 (.024)	.043 (.025)
PP3→PP4	.321 (.024)***	.311 (.024)***	PP3→EM4	.098 (.029)***	.101 (.024)***
PP4→PP5	.347 (.024)***	.371 (.025)***	PP4→EM5	.114 (.024)***	.123 (.026)***
PP5→PP6	.328 (.023)***	.351 (.024)***	PP5→EM6	.063 (.021)***	.064 (.021)***
Trait correlation	.528 (.029)***	.587 (.025)***			
**Model: EM-HYP**					
EM1→EM2	.100 (.025)***	.117 (.028)***	EM1→HYP2	-.083 (.021)***	-.097 (.025)***
EM2→EM3	.264 (.030)***	.256 (.028)***	EM2→HYP3	.010 (.027)	.010 (.028)
EM3→EM4	.322 (.025)***	.325 (.024)***	EM3→HYP4	.082 (.024)***	.082 (.024)***
EM4→EM5	.345 (.024)***	.350 (.023)***	EM4→HYP5	.045 (.025)	.046 (.025)
EM5→EM6	.430 (.022)***	.435 (.021)***	EM5→HYP6	.129 (.023)***	.132 (.023)***
HYP1→HYP2	.221 (.022)***	.246 (.023)***	HYP1→EM2	-.039 (.023)	-.043 (.025)
HYP2→HYP3	.304 (.025)***	.316 (.026)***	HYP2→EM3	.049 (.025)	.047 (.024)
HYP3→HYP4	.389 (.031)***	.358 (.028)***	HYP3→EM4	.066 (.027)*	.061 (.025)*
HYP4→HYP5	.393 (.033)***	.409 (.032)***	HYP4→EM5	.120 (.025)***	.123 (.026)***
HYP5→HYP6	.372 (.031)***	.376 (.031)***	HYP5→EM6	.049 (.022)*	.049 (.022)*
Trait correlation	.315 (.034)***	.387 (.031)***			
**Model: EM-CON**					
EM1→EM2	.101 (.024)***	.117 (.028)***	EM1→CON2	-.033 (.022)	-.036 (.028)
EM2→EM3	.277 (.030)***	.268 (.027)***	EM2→CON3	.045 (.027)	.046 (.028)
EM3→EM4	.319 (.025)***	.322 (.025)***	EM3→CON4	.008 (.025)	.007 (.024)
EM4→EM5	.377 (.025)***	.382 (.024)***	EM4→CON5	.088 (.027)***	.093 (.028)***
EM5→EM6	.428 (.022)***	.435 (.021)***	EM5→CON6	.040 (.025)	.039 (.025)
CON1→CON2	.224 (.022)***	.245 (.025)***	CON1→EM2	.059 (.022)**	.067 (.024)**
CON2→CON3	.217 (.031)***	.233 (.031)***	CON2→EM3	.029 (.025)	.029 (.025)
CON3→CON4	.293 (.034)***	.262 (.032)***	CON3→EM4	.102 (.027)***	.097 (.027)***
CON4→CON5	.214 (.041)***	.241 (.041)***	CON4→EM5	.034 (.026)	.036 (.027)
CON5→CON6	.309 (.039)***	.285 (.038)***	CON5→EM6	.058 (.025)*	.056 (.024)*
Trait correlation	.409 (.033)***	.485 (.031)***			
**Model: PP-HYP**					
PP1→PP2	.117 (.025)***	.114 (.024)***	PP1→HYP2	-.073 (.022)***	-.077 (.023)***
PP2→PP3	.185 (.026)***	.184 (.026)***	PP2→HYP3	.007 (.022)	.008 (.023)
PP3→PP4	.309 (.023)***	.299 (.022)***	PP3→HYP4	.079 (.023)***	.080 (.023)***
PP4→PP5	.341 (.022)***	.366 (.023)***	PP4→HYP5	.059 (.023)**	.064 (.024)**
PP5→PP6	.346 (.023)***	.369 (.024)***	PP5→HYP6	.053 (.021)*	.055 (.021)*
HYP1→HYP2	.204 (.022)***	.229 (.023)***	HYP1→PP2	.016 (.024)	.017 (.025)
HYP2→HYP3	.291 (.026)***	.302 (.027)***	HYP2→PP3	.078 (.024)***	.072 (.023)***
HYP3→HYP4	.389 (.031)***	.356 (.028)***	HYP3→PP4	.103 (.026)***	.089 (.023)***
HYP4→HYP5	.392 (.032)***	.407 (.031)***	HYP4→PP5	.067 (.023)***	.068 (.023)***
HYP5→HYP6	.407 (.029)***	.414 (.029)***	HYP5→PP6	.065 (.021)***	.068 (.022)***
Trait correlation	.499 (.027)***	.497 (.026)***			
**Model: PP-CON**					
PP1→PP2	.106 (.025)***	.103 (.024)***	PP1→CON2	-.089 (.023)***	-.088 (.024)***
PP2→PP3	.188 (.026)***	.187 (.026)***	PP2→CON3	.027 (.025)	.030 (.027)
PP3→PP4	.304 (.024)***	.294 (.023)***	PP3→CON4	.069 (.024)***	.067 (.023)***
PP4→PP5	.347 (.023)***	.373 (.024)***	PP4→CON5	.111 (.029)***	.126 (.033)***
PP5→PP6	.337 (.023)***	.362 (.023)***	PP5→CON6	.094 (.028)***	.091 (.027)***
CON1→CON2	.223 (.022)***	.244 (.024)***	CON1→PP2	.063 (.023)**	.068 (.025)**
CON2→CON3	.216 (.029)***	.235 (.029)***	CON2→PP3	.056 (.023)*	.055 (.022)*
CON3→CON4	.285 (.033)***	.253 (.031)***	CON3→PP4	.128 (.033)***	.112 (.029)***
CON4→CON5	.224 (.040)***	.251 (.039)***	CON4→PP5	.069 (.024)***	.073 (.025)***
CON5→CON6	.308 (.037)***	.284 (.036)***	CON5→PP6	.097 (.028)***	.099 (.029)***
Trait correlation	.476 (.030)***	.540 (.027)***			
**Model: HYP-CON**					
CON1→CON2	.200 (.022)***	.218 (.024)***	CON1→HYP2	.032 (.019)	.037 (.022)
CON2→CON3	.187 (.030)***	.205 (.031)***	CON2→HYP3	.047 (.022)*	.052 (.024)*
CON3→CON4	.274 (.033)***	.242 (.031)***	CON3→HYP4	.065 (.027)*	.061 (.025)*
CON4→CON5	.219 (.037)***	.247 (.037)***	CON4→HYP5	.029 (.025)	.031 (.027)
CON5→CON6	.219 (.037)***	.279 (.036)***	CON5→HYP6	.099 (.029)***	.097 (.028)***
HYP1→HYP2	.203 (.023)***	.226 (.037)***	HYP1→CON2	.014 (.024)	.015 (.025)
HYP2→HYP3	.271 (.025)***	.285 (.026)***	HYP2→CON3	.041 (.026)	.042 (.027)
HYP3→HYP4	.362 (.031)***	.332 (.027)***	HYP3→CON4	.022 (.031)	.019 (.027)
HYP4→HYP5	.375 (.031)***	.338 (.031)***	HYP4→CON5	.077 (.030)**	.082 (.031)**
HYP5→HYP6	.364 (.030)***	.368 (.030)***	HYP5→CON6	.075 (.028)**	.071 (.027)**
Trait correlation	.650 (.022)***	.696 (.016)***			

*Note*:

*p < .05;

**p < .01;

***p < .001;

B cohort: Baby cohort; K cohort: Kindergarten cohort; β: Standardised linear regression coefficient; S.E.: Standard error; EM: Emotional symptoms; PP: Peer problems; HYP: Hyperactivity; CON: Conduct problems; Trait correlation: Correlation between the random-intercepts; p-values based on the unstandardised solution; statistical significance did not vary between the standardised and the unstandardised solutions.

#### Robustness checks- including covariates

To assess the robustness of the dynamic within-child findings, the three covariates were introduced as predictors of the random intercepts in the bivariate multigroup RI-CLPMs. The results of the robustness checks by adding covariates did not reveal any substantial differences between the unconditional multigroup RI-CLPMs and the conditional models (see S1–S6 Tables). On average, parental mental health difficulties were generally predicting greater child internalising and externalising symptoms. Greater household income was a negative predictor of emotional symptoms, peer and conduct problems but did not predict hyperactivity. Female children had fewer conduct problems, hyperactivity, and peer problems but slightly raised emotional symptoms over time.

## Discussion

Given the inconsistencies in the findings of previous studies regarding the co-occurrence between internalising and externalising mental health symptoms, the current study examined the longitudinal interplay between different types of internalising and externalising symptoms. This is an important area of research because it can reveal new insights into how symptoms within the same typology and across different typologies relate to each other as children grow. The study examined whether symptoms co-occurred within the same children, whether general mental ill-health personality traits could account for the shared variance, and whether issues in one area lead to problems in the other. In the section below, the current findings are unpacked and linked with previous evidence.

The models had excellent fit to the data, suggesting, in line with state-trait theory [41,52], that children’s mental health symptoms are characterised both by general stability over time and increasing or decreasing changes as children develop. The advantage of the state-trait modelling approach is that it investigates how *transient* changes in one domain of symptoms are predictive of changes in the other domain, rather than conflating stable trait-level variance with within-child changes [40]. This means that there is a clear developmental focus on the processes that occur within the children’s developing selves, rather than focusing on between-child differences.

Following the state-trait approach, the results of the cross-cohort comparison of the structural relations revealed no statistically significant differences in the unstandardised regression coefficients between the two cohorts. Seemingly, this finding might not be surprising since the kindergarten and baby cohorts come from Australia. Although this is purely exploratory at this point, the additional insight is that the structural relations generalise over two different observational periods (2004–2014 vs. 2008–2018). This shows that the developmental relations between the different symptoms remain stable in the passage of calendar years in the population, which is a novel insight that has not been previously shown. This is quite an interesting finding since previous studies, which employed independent samples, have reached different conclusions regarding the directional relations between internalising and externalising symptoms [e.g., 6,11,15].

Moving on to the second research hypothesis, the results of the modelling revealed positive associations on most occasions. An exception to this general positive association is that some between-domain (i.e., peer problems- hyperactivity; emotional symptoms- hyperactivity) effects at age four years were found to be negative (see Table 4). Some sporadic negative effects have been reported in past studies, too [23,25], though these are considered here to reflect developmental instabilities within children. Overall, the positive associations between the different aspects of internalising and externalising domains are something that has been noted in the literature [9,10,25,63] and is compatible with accounts of heterotypic co-occurrence in internalising and externalising symptoms [11].

Additionally, the present study contributes new insights since it illustrates several reciprocal links between and within the different domains of mental health symptoms. First, clear positive reciprocal relations were found within the internalising domain, whereby emotional symptoms and peer problems were bidirectionally associated over time from age 8 years onwards. This confirms the dynamic mutualism in the internalising domain [7,16]. Second, sporadic and inconsistent positive effects were observed between emotional symptoms and conduct problems, shedding doubt on the co-occurrence between emotional symptoms and conduct problems. The relations between emotional symptoms and hyperactivity were characterised as reciprocal only in the transitions from middle childhood to early adolescence and from early adolescence to mid-adolescence.

In contrast, the relations of peer problems with conduct problems and hyperactivity were consistently bidirectional from age 8 years onwards. This finding suggests that consistent evidence of co-occurrence across domains in this general population sample might stem from peer problems rather than emotional difficulties. Of particular note is that within the domain of externalising symptoms modest reciprocal relations were identified only in between early and middle adolescence, between ages 12 and 14. This suggests that the co-occurrence of the externalising symptoms occurs only after transitioning to early adolescence. Adolescence is a developmental stage fraught with developmental challenges for children’s mental health and well-being [64,65], and this might be the reason why these mutually reinforcing effects are particularly observed in this period. Otherwise, conduct problems, seemed to predict small increases in hyperactivity between early childhood (age 6) and the beginning of early adolescence (age 10). This latter finding is counterintuitive since past evidence has suggested that hyperactivity and/or inattention might predict worse conduct problems [32–34], but might be an outcome of the partially strict invariance of the conduct problems.

Finally, the co-occurrence between and within the internalising and externalising domains has been attributed in part to a general common psychopathology factor [16,18]. In the state-trait approach adopted, this general psychopathology factor is reflected on the correlation between the random intercepts that represent the traits of internalising and externalising domains [37]. Although the trait-level correlations were all positive and reached statistical significance as expected [10,26], these correlations were rather moderate-to-strong (explained variance range: 10%- 48%), suggesting the existence of shared variance at the trait level, on average. However, the magnitude of these trait-level correlations was nowhere near unity, which indicates that a single underlying trait factor does not fully explain the associations [42] within and between the different facets of internalising and externalising mental health symptoms over the course of development. This latter conclusion needs to be further verified in future longitudinal studies.

In general terms, the mostly positive and reciprocal associations between the different symptoms of the internalising and externalising domains contradict previous evidence in favour of unidirectional effects [10,25,63], but support some other evidence in favour of bidirectionality in children’s mental health development [13,27]. This suggests that the theoretical view of co-occurrence [7] indicating that a preponderance of externalising predicts subsequent internalising does not hold much merit for these populations. The dynamic mutualism theoretical view [18,19] of co-occurrence seems to be a more promising explanation of the co-occurrence. This mutualism is supported by the reciprocal nature of the longitudinal association and is mostly manifesting in the transitions to adolescence and from middle childhood onwards. This pattern of mutualism appears to be consistent with the accounts of increased symptoms and particularly in the adolescent years [1–4].

### Strengths and limitations

The present study has several strengths, such as the representative samples, the cross-cohort comparative approach, the invariant measures with good psychometric properties, and the trait-state approach. Nevertheless, some limitations should be noted. For instance, the SDQ measure is a screening tool designed for the general population of children and does not necessarily accurately capture all aspects of children’s mental health [66]. Additionally, the data predate the COVID-19 pandemic and may, thus, not accurately reflect the current state of children’s mental health. Finally, a replication of the current findings with teacher- and self-reports, as well as with more refined measures of children’s mental health, is needed.

### Implications for theory and practice

The current findings contribute to the theory of children’s mental health development by clearly examining the developmental cascades within and between the internalising and externalising mental health domains. The results indicate that the transitions from middle childhood to early adolescence and to mid-adolescence are critical periods for children’s mental health development since most of the bidirectional associations occurred in these stages. On the practical side of things, the findings would suggest that children facing emotional difficulties typically have troubled peer relationships. However, children with higher-than-usual hyperactivity do not necessarily exhibit conduct problems (e.g., bullying or stealing), except in the transition from early to middle adolescence, between ages 12 and 14 years. Provided the consistent bidirectionalities between peer problems and externalising symptoms, professionals should be aware that potentially troubled peer relationships are associated with conduct problems and hyperactivity, and vice versa. Interventions are needed particularly in adolescence to prevent exacerbating mental health symptoms due to the tendency for dynamic mutualism between and within the two domains of psychopathology. Children and adolescents could be taught coping skills and socio-emotional skills to become more resilient against the development of increased mental health symptoms. However, educators, parents, and practitioners also have a role to play by being vigilant in identifying children and adolescents who need additional support. Finally, researchers need to raise the awareness that the manifestation of childhood and adolescent mental health symptoms is a fluid process that takes time and may be reversed due to potential discontinuities and particular life experiences [67]. Hence, long-term monitoring of children’s and adolescents’ mental health status is recommended in schools and communities.

## Conclusion

The study revealed that the links between different aspects of internalising and externalising mental health symptoms are more complicated than previously believed. Moderate-to-strong within-child reciprocal relations were observed within the domain of internalising symptoms, but not within the externalising domain, except between 12 and 14 years. Sporadic cross-domain propagation of mental health difficulties was observed between emotional symptoms, hyperactivity, and conduct problems. Consistent mutual reinforcement was observed between peer problems and the indices of the externalising symptoms. These relations were, on average, robust across two different cohorts of children born four years apart in Australia. Overall, the present analyses provide strong and consistent evidence regarding the co-occurrence of the internalising and externalising symptoms of mental health and test hypotheses about the temporal precedence of specific symptoms. The study suggests that a dynamic mutualism explanation of the co-occurrence of mental health symptoms holds more merit from middle childhood onwards and particularly in adolescence. In other words, symptoms were initially weakly associated but the different symptoms started to causally predict each other later on.

## Supporting information

S1 Table Robustness check 1 RI-CLPM EM-PP. (DOCX)

S2 Table Robustness check 2 RI-CLPM EM-HYP. (DOCX)

S3 Table Robustness check 3 RI-CLPM EM-CON. (DOCX)

S4 Table Robustness check 4 RI-CLPM PP-HYP. (DOCX)

S5 Table Robustness check 5 RI-CLPM PP-CON. (DOCX)

S6 Table Robustness check 6 RI-CLPM HYP-CON. (DOCX)
