# Supplementary material for: Dynamic interplay of developing internalising and externalising mental health from early childhood to mid-adolescence: Teasing apart trait, state, and cross-cohort effects
Source: PLoS One. 2024 Jul 10;19(7):e0306978. doi: 10.1371/journal.pone.0306978 (PMC11236104; doi:10.1371/journal.pone.0306978)
Supplement: S1 Table — (DOCX) [file pone.0306978.s001.docx]

Table S1. Standardised parameter estimates for robustness check 1- Bivariate RI-CLPM of emotional symptoms and peer problems

β estimate S.E. β/S.E. Two-tailed p-value

**Baby cohort**

OEM2 ON

OEM1 0.092 0.024 3.891 0.000

OPP1 0.030 0.023 1.339 0.181

OEM3 ON

OEM2 0.277 0.030 9.168 0.000

OPP2 0.038 0.023 1.651 0.099

OEM4 ON

OEM3 0.325 0.025 12.766 0.000

OPP3 0.094 0.023 4.118 0.000

OEM5 ON

OEM4 0.351 0.026 13.707 0.000

OPP4 0.117 0.024 4.935 0.000

OEM6 ON

OEM5 0.429 0.021 20.158 0.000

OPP5 0.075 0.021 3.572 0.000

OPP2 ON

OPP1 0.096 0.024 3.998 0.000

OEM1 0.027 0.021 1.241 0.215

OPP3 ON

OPP2 0.181 0.026 6.953 0.000

OEM2 0.104 0.024 4.263 0.000

OPP4 ON

OPP3 0.321 0.024 13.397 0.000

OEM3 0.109 0.024 4.519 0.000

OPP5 ON

OPP4 0.349 0.023 14.856 0.000

OEM4 0.099 0.021 4.690 0.000

OPP6 ON

OPP5 0.333 0.023 14.273 0.000

OEM5 0.137 0.021 6.627 0.000

TPP ON

SEX -0.105 0.024 -4.369 0.000

INCGROUP -0.067 0.023 -2.961 0.003

MH 0.263 0.030 8.851 0.000

TEM ON

SEX 0.089 0.029 3.089 0.002

INCGROUP -0.067 0.022 -3.052 0.002

MH 0.336 0.037 9.124 0.000

TPP WITH

TEM 0.495 0.032 15.660 0.000

**Kindergarten cohort**

OEM2 ON

OEM1 0.108 0.028 3.889 0.000

OPP1 0.032 0.024 1.350 0.177

OEM3 ON

OEM2 0.269 0.028 9.523 0.000

OPP2 0.039 0.024 1.635 0.102

OEM4 ON

OEM3 0.327 0.024 13.376 0.000

OPP3 0.097 0.023 4.150 0.000

OEM5 ON

OEM4 0.352 0.024 14.730 0.000

OPP4 0.126 0.025 4.964 0.000

OEM6 ON

OEM5 0.435 0.020 21.335 0.000

OPP5 0.076 0.021 3.571 0.000

OPP2 ON

OPP1 0.095 0.024 3.930 0.000

OEM1 0.029 0.024 1.244 0.214

OPP3 ON

OPP2 0.180 0.026 6.892 0.000

OEM2 0.098 0.024 4.143 0.000

OPP4 ON

OPP3 0.310 0.023 13.338 0.000

OEM3 0.102 0.023 4.524 0.000

OPP5 ON

OPP4 0.373 0.025 14.852 0.000

OEM4 0.099 0.021 4.684 0.000

OPP6 ON

OPP5 0.356 0.024 14.958 0.000

OEM5 0.146 0.021 6.822 0.000

TPP ON

SEX -0.097 0.019 -4.974 0.000

INCGROUP -0.042 0.021 -2.014 0.044

MH 0.330 0.026 12.484 0.000

TEM ON

SEX 0.073 0.022 3.240 0.001

INCGROUP -0.062 0.022 -2.805 0.005

MH 0.436 0.025 17.733 0.000

TPP WITH

TEM 0.536 0.029 18.670 0.000

ON: Regressed on; WITH: Correlation; β: Standardised linear regression coefficient; SEX: Female vs. male; INCGROUP: Income groups; MH: Average of paternal and maternal Kessler 6 scores; OPP: Peer problems occasion-specific residual at time t; OEM: Emotional symptoms occasion-specific residual at time t; TPP: Random-intercept of Peer problems; TEM: Random-intercept of emotional symptoms
