# Supplementary material for: Dynamic interplay of developing internalising and externalising mental health from early childhood to mid-adolescence: Teasing apart trait, state, and cross-cohort effects
Source: PLoS One. 2024 Jul 10;19(7):e0306978. doi: 10.1371/journal.pone.0306978 (PMC11236104; doi:10.1371/journal.pone.0306978)
Supplement: S2 Table — (DOCX) [file pone.0306978.s002.docx]

Table S2. Standardised parameter estimates for robustness check 2- Bivariate RI-CLPM of emotional symptoms and hyperactivity

β estimate S.E. β/S.E. Two-tailed p-value

**Baby cohort**

OEM2 ON

OEM1 0.099 0.024 4.126 0.000

OHYP1 -0.058 0.022 -2.589 0.010

OEM3 ON

OEM2 0.275 0.029 9.409 0.000

OHYP2 0.045 0.025 1.824 0.068

OEM4 ON

OEM3 0.328 0.025 13.364 0.000

OHYP3 0.070 0.026 2.682 0.007

OEM5 ON

OEM4 0.344 0.024 14.177 0.000

OHYP4 0.130 0.025 5.244 0.000

OEM6 ON

OEM5 0.417 0.022 18.945 0.000

OHYP5 0.077 0.022 3.512 0.000

OHYP2 ON

OHYP1 0.225 0.021 10.884 0.000

OEM1 -0.095 0.021 -4.549 0.000

OHYP3 ON

OHYP2 0.314 0.025 12.749 0.000

OEM2 0.004 0.026 0.174 0.862

OHYP4 ON

OHYP3 0.400 0.030 13.376 0.000

OEM3 0.081 0.024 3.408 0.001

OHYP5 ON

OHYP4 0.395 0.032 12.503 0.000

OEM4 0.053 0.024 2.236 0.025

OHYP6 ON

OHYP5 0.370 0.030 12.362 0.000

OEM5 0.142 0.022 6.355 0.000

THYP ON

SEX -0.249 0.023 -11.023 0.000

INCGROUP -0.027 0.024 -1.121 0.262

MH 0.151 0.024 6.199 0.000

TEM ON

SEX 0.099 0.028 3.500 0.000

INCGROUP -0.066 0.022 -3.030 0.002

MH 0.332 0.036 9.230 0.000

THYP WITH

TEM 0.328 0.036 9.171 0.000

**Kindergarten cohort**

OEM2 ON

OEM1 0.115 0.028 4.136 0.000

OHYP1 -0.064 0.025 -2.580 0.010

OEM3 ON

OEM2 0.265 0.027 9.746 0.000

OHYP2 0.043 0.024 1.822 0.068

OEM4 ON

OEM3 0.331 0.024 13.938 0.000

OHYP3 0.065 0.024 2.644 0.008

OEM5 ON

OEM4 0.347 0.023 15.074 0.000

OHYP4 0.134 0.025 5.268 0.000

OEM6 ON

OEM5 0.423 0.021 19.783 0.000

OHYP5 0.077 0.022 3.473 0.001

OHYP2 ON

OHYP1 0.251 0.022 11.529 0.000

OEM1 -0.111 0.024 -4.523 0.000

OHYP3 ON

OHYP2 0.329 0.026 12.760 0.000

OEM2 0.005 0.027 0.173 0.862

OHYP4 ON

OHYP3 0.365 0.027 13.489 0.000

OEM3 0.081 0.023 3.438 0.001

OHYP5 ON

OHYP4 0.413 0.031 13.471 0.000

OEM4 0.054 0.024 2.226 0.026

OHYP6 ON

OHYP5 0.372 0.030 12.528 0.000

OEM5 0.145 0.023 6.286 0.000

THYP ON

SEX -0.301 0.018 -16.460 0.000

INCGROUP -0.025 0.022 -1.126 0.260

MH 0.228 0.022 10.534 0.000

TEM ON

SEX 0.080 0.022 3.645 0.000

INCGROUP -0.065 0.022 -3.013 0.003

MH 0.431 0.024 18.079 0.000

THYP WITH

TEM 0.358 0.032 11.040 0.000

ON: Regressed on; WITH: Correlation; β: Standardised linear regression coefficient; SEX: Female vs. male; INCGROUP: Income groups; MH: Average of paternal and maternal Kessler 6 scores; OHYP: Hyperactivity occasion-specific residual at time t; OEM: Emotional symptoms occasion-specific residual at time t; THYP: Random-intercept of Hyperactivity; TEM: Random-intercept of emotional symptoms
