# Supplementary material for: Dynamic interplay of developing internalising and externalising mental health from early childhood to mid-adolescence: Teasing apart trait, state, and cross-cohort effects
Source: PLoS One. 2024 Jul 10;19(7):e0306978. doi: 10.1371/journal.pone.0306978 (PMC11236104; doi:10.1371/journal.pone.0306978)
Supplement: S3 Table — (DOCX) [file pone.0306978.s003.docx]

Table S3. Standardised parameter estimates for robustness check 3- Bivariate RI-CLPM of emotional symptoms and conduct problems

β estimate S.E. β/S.E. Two-tailed p-value

Baby cohort

OEM2 ON

OEM1 0.100 0.023 4.276 0.000

OCON1 0.044 0.021 2.044 0.041

OEM3 ON

OEM2 0.283 0.029 9.597 0.000

OCON2 0.026 0.025 1.045 0.296

OEM4 ON

OEM3 0.321 0.024 13.168 0.000

OCON3 0.104 0.027 3.828 0.000

OEM5 ON

OEM4 0.376 0.025 14.777 0.000

OCON4 0.041 0.026 1.623 0.105

OEM6 ON

OEM5 0.421 0.022 19.178 0.000

OCON5 0.073 0.025 2.974 0.003

OCON2 ON

OCON1 0.214 0.022 9.686 0.000

OEM1 -0.046 0.022 -2.102 0.036

OCON3 ON

OCON2 0.212 0.030 7.022 0.000

OEM2 0.044 0.027 1.612 0.107

OCON4 ON

OCON3 0.295 0.034 8.747 0.000

OEM3 0.012 0.026 0.462 0.644

OCON5 ON

OCON4 0.224 0.040 5.650 0.000

OEM4 0.090 0.027 3.339 0.001

OCON6 ON

OCON5 0.324 0.038 8.522 0.000

OEM5 0.044 0.025 1.734 0.083

TCON ON

SEX -0.100 0.025 -3.957 0.000

INCGROUP -0.069 0.022 -3.061 0.002

MH 0.207 0.028 7.314 0.000

TEM ON

SEX 0.091 0.029 3.162 0.002

INCGROUP -0.065 0.022 -2.993 0.003

MH 0.332 0.036 9.128 0.000

TCON WITH

TEM 0.375 0.036 10.477 0.000

**Kindergarten cohort**

OEM2 ON

OEM1 0.116 0.027 4.313 0.000

OCON1 0.050 0.024 2.072 0.038

OEM3 ON

OEM2 0.273 0.027 10.061 0.000

OCON2 0.026 0.025 1.056 0.291

OEM4 ON

OEM3 0.325 0.024 13.429 0.000

OCON3 0.099 0.027 3.717 0.000

OEM5 ON

OEM4 0.379 0.024 15.943 0.000

OCON4 0.044 0.027 1.646 0.100

OEM6 ON

OEM5 0.429 0.021 20.408 0.000

OCON5 0.071 0.024 2.964 0.003

OCON2 ON

OCON1 0.234 0.024 9.657 0.000

OEM1 -0.051 0.024 -2.132 0.033

OCON3 ON

OCON2 0.228 0.031 7.336 0.000

OEM2 0.045 0.028 1.624 0.104

OCON4 ON

OCON3 0.264 0.031 8.442 0.000

OEM3 0.011 0.024 0.461 0.644

OCON5 ON

OCON4 0.251 0.040 6.287 0.000

OEM4 0.095 0.028 3.418 0.001

OCON6 ON

OCON5 0.299 0.037 8.142 0.000

OEM5 0.042 0.025 1.716 0.086

TCON ON

SEX -0.135 0.020 -6.623 0.000

INCGROUP -0.038 0.019 -1.962 0.050

MH 0.295 0.025 11.824 0.000

TEM ON

SEX 0.071 0.022 3.181 0.001

INCGROUP -0.062 0.022 -2.825 0.005

MH 0.435 0.025 17.569 0.000

TCON WITH

TEM 0.433 0.033 13.139 0.000

ON: Regressed on; WITH: Correlation; β: Standardised linear regression coefficient; SEX: Female vs. male; INCGROUP: Income groups; MH: Average of paternal and maternal Kessler 6 scores; OCON: Conduct problems occasion-specific residual at time t; OEM: Emotional symptoms occasion-specific residual at time t; TCON: Random-intercept of conduct problems; TEM: Random-intercept of emotional symptoms
