# Supplementary material for: Dynamic interplay of developing internalising and externalising mental health from early childhood to mid-adolescence: Teasing apart trait, state, and cross-cohort effects
Source: PLoS One. 2024 Jul 10;19(7):e0306978. doi: 10.1371/journal.pone.0306978 (PMC11236104; doi:10.1371/journal.pone.0306978)
Supplement: S4 Table — (DOCX) [file pone.0306978.s004.docx]

Table S4. Standardised parameter estimates for robustness check 4- Bivariate RI-CLPM of hyperactivity and peer problems

β estimate S.E. β/S.E. Two-tailed p-value

**Baby cohort**

OPP2 ON

OPP1 0.105 0.024 4.312 0.000

OHYP1 0.010 0.024 0.406 0.685

OPP3 ON

OPP2 0.179 0.026 6.837 0.000

OHYP2 0.083 0.024 3.463 0.001

OPP4 ON

OPP3 0.310 0.023 13.587 0.000

OHYP3 0.109 0.026 4.244 0.000

OPP5 ON

OPP4 0.346 0.022 15.665 0.000

OHYP4 0.068 0.023 3.000 0.003

OPP6 ON

OPP5 0.350 0.023 15.314 0.000

OHYP5 0.074 0.021 3.594 0.000

OHYP2 ON

OHYP1 0.209 0.021 10.017 0.000

OPP1 -0.073 0.021 -3.415 0.001

OHYP3 ON

OHYP2 0.293 0.026 11.482 0.000

OPP2 0.009 0.021 0.397 0.691

OHYP4 ON

OHYP3 0.389 0.030 12.834 0.000

OPP3 0.081 0.022 3.634 0.000

OHYP5 ON

OHYP4 0.387 0.031 12.456 0.000

OPP4 0.065 0.022 2.917 0.004

OHYP6 ON

OHYP5 0.405 0.028 14.552 0.000

OPP5 0.057 0.020 2.766 0.006

THYP ON

SEX -0.238 0.022 -10.577 0.000

INCGROUP -0.026 0.024 -1.084 0.278

MH 0.154 0.025 6.278 0.000

TPP ON

SEX -0.093 0.024 -3.922 0.000

INCGROUP -0.063 0.023 -2.802 0.005

MH 0.257 0.029 8.915 0.000

THYP WITH

TPP 0.471 0.028 16.564 0.000

**Kindergarten cohort**

OPP2 ON

OPP1 0.103 0.024 4.272 0.000

OHYP1 0.010 0.025 0.405 0.686

OPP3 ON

OPP2 0.178 0.026 6.842 0.000

OHYP2 0.077 0.022 3.449 0.001

OPP4 ON

OPP3 0.300 0.022 13.689 0.000

OHYP3 0.094 0.022 4.235 0.000

OPP5 ON

OPP4 0.371 0.023 15.861 0.000

OHYP4 0.069 0.023 2.995 0.003

OPP6 ON

OPP5 0.374 0.023 16.092 0.000

OHYP5 0.077 0.021 3.649 0.000

OHYP2 ON

OHYP1 0.233 0.022 10.432 0.000

OPP1 -0.076 0.022 -3.410 0.001

OHYP3 ON

OHYP2 0.307 0.027 11.472 0.000

OPP2 0.010 0.024 0.397 0.691

OHYP4 ON

OHYP3 0.353 0.027 13.116 0.000

OPP3 0.082 0.022 3.719 0.000

OHYP5 ON

OHYP4 0.403 0.030 13.402 0.000

OPP4 0.071 0.024 2.949 0.003

OHYP6 ON

OHYP5 0.410 0.028 14.776 0.000

OPP5 0.058 0.021 2.772 0.006

THYP ON

SEX -0.285 0.019 -15.296 0.000

INCGROUP -0.028 0.022 -1.238 0.216

MH 0.229 0.022 10.455 0.000

TPP ON

SEX -0.088 0.019 -4.670 0.000

INCGROUP -0.044 0.020 -2.186 0.029

MH 0.326 0.026 12.346 0.000

THYP WITH

TPP 0.444 0.028 15.945 0.000

ON: Regressed on; WITH: Correlation; β: Standardised linear regression coefficient; SEX: Female vs. male; INCGROUP: Income groups; MH: Average of paternal and maternal Kessler 6 scores; OHYP: Hyperactivity occasion-specific residual at time t; OPP: Peer problems occasion-specific residual at time t; THYP: Random-intercept of hyperactivity; TPP: Random-intercept of peer problems
