# Supplementary material for: Dynamic interplay of developing internalising and externalising mental health from early childhood to mid-adolescence: Teasing apart trait, state, and cross-cohort effects
Source: PLoS One. 2024 Jul 10;19(7):e0306978. doi: 10.1371/journal.pone.0306978 (PMC11236104; doi:10.1371/journal.pone.0306978)
Supplement: S5 Table — (DOCX) [file pone.0306978.s005.docx]

Table S5. Standardised parameter estimates for robustness check 5- Bivariate RI-CLPM of peer problems and conduct problems

β estimate S.E. β/S.E. Two-tailed p-value

**Baby cohort**

OPP2 ON

OPP1 0.095 0.024 3.921 0.000

OCON1 0.050 0.023 2.172 0.030

OPP3 ON

OPP2 0.181 0.026 7.001 0.000

OCON2 0.052 0.022 2.338 0.019

OPP4 ON

OPP3 0.303 0.024 12.880 0.000

OCON3 0.133 0.032 4.112 0.000

OPP5 ON

OPP4 0.349 0.022 15.669 0.000

OCON4 0.074 0.024 3.125 0.002

OPP6 ON

OPP5 0.340 0.023 14.864 0.000

OCON5 0.105 0.028 3.790 0.000

OCON2 ON

OCON1 0.212 0.022 9.832 0.000

OPP1 -0.100 0.023 -4.282 0.000

OCON3 ON

OCON2 0.211 0.029 7.406 0.000

OPP2 0.023 0.025 0.944 0.345

OCON4 ON

OCON3 0.288 0.033 8.796 0.000

OPP3 0.070 0.024 2.947 0.003

OCON5 ON

OCON4 0.231 0.039 5.984 0.000

OPP4 0.116 0.029 4.055 0.000

OCON6 ON

OCON5 0.319 0.036 8.770 0.000

OPP5 0.101 0.027 3.665 0.000

TCON ON

SEX -0.098 0.025 -3.946 0.000

INCGROUP -0.069 0.022 -3.066 0.002

MH 0.213 0.029 7.452 0.000

TPP ON

SEX -0.098 0.024 -4.146 0.000

INCGROUP -0.064 0.022 -2.858 0.004

MH 0.260 0.029 8.968 0.000

TCON WITH

TPP 0.433 0.032 13.522 0.000

**Kindergarten cohort**

OPP2 ON

OPP1 0.093 0.024 3.894 0.000

OCON1 0.054 0.025 2.173 0.030

OPP3 ON

OPP2 0.181 0.026 7.013 0.000

OCON2 0.051 0.022 2.360 0.018

OPP4 ON

OPP3 0.292 0.023 12.805 0.000

OCON3 0.116 0.028 4.074 0.000

OPP5 ON

OPP4 0.375 0.024 15.876 0.000

OCON4 0.078 0.025 3.131 0.002

OPP6 ON

OPP5 0.365 0.023 15.760 0.000

OCON5 0.107 0.028 3.797 0.000

OCON2 ON

OCON1 0.232 0.024 9.748 0.000

OPP1 -0.099 0.023 -4.221 0.000

OCON3 ON

OCON2 0.230 0.029 7.907 0.000

OPP2 0.026 0.027 0.945 0.344

OCON4 ON

OCON3 0.254 0.030 8.512 0.000

OPP3 0.068 0.023 2.984 0.003

OCON5 ON

OCON4 0.257 0.038 6.843 0.000

OPP4 0.131 0.032 4.096 0.000

OCON6 ON

OCON5 0.294 0.035 8.403 0.000

OPP5 0.097 0.027 3.656 0.000

TCON ON

SEX -0.124 0.021 -6.021 0.000

INCGROUP -0.040 0.019 -2.052 0.040

MH 0.296 0.025 11.694 0.000

TPP ON

SEX -0.094 0.019 -4.937 0.000

INCGROUP -0.042 0.020 -2.062 0.039

MH 0.328 0.027 12.336 0.000

TCON WITH

TPP 0.484 0.028 17.324 0.000

ON: Regressed on; WITH: Correlation; β: Standardised linear regression coefficient; SEX: Female vs. male; INCGROUP: Income groups; MH: Average of paternal and maternal Kessler 6 scores; OCON: Conduct problems occasion-specific residual at time t; OPP: Peer problems occasion-specific residual at time t; TCON: Random-intercept of conduct problems; TPP: Random-intercept of peer problems
