# Supplementary material for: Dynamic interplay of developing internalising and externalising mental health from early childhood to mid-adolescence: Teasing apart trait, state, and cross-cohort effects
Source: PLoS One. 2024 Jul 10;19(7):e0306978. doi: 10.1371/journal.pone.0306978 (PMC11236104; doi:10.1371/journal.pone.0306978)
Supplement: S6 Table — (DOCX) [file pone.0306978.s006.docx]

Table S6. Standardised parameter estimates for robustness check 6- Bivariate RI-CLPM of hyperactivity and conduct problems

β estimate S.E. β/S.E. Two-tailed p-value

**Baby cohort**

OHYP2 ON

OHYP1 0.206 0.022 9.389 0.000

OCON1 0.027 0.018 1.446 0.148

OHYP3 ON

OHYP2 0.270 0.024 11.153 0.000

OCON2 0.041 0.022 1.858 0.063

OHYP4 ON

OHYP3 0.360 0.030 12.034 0.000

OCON3 0.064 0.027 2.381 0.017

OHYP5 ON

OHYP4 0.368 0.030 12.284 0.000

OCON4 0.036 0.025 1.451 0.147

OHYP6 ON

OHYP5 0.360 0.029 12.407 0.000

OCON5 0.107 0.028 3.809 0.000

OCON2 ON

OCON1 0.192 0.022 8.847 0.000

OHYP1 0.003 0.023 0.111 0.912

OCON3 ON

OCON2 0.185 0.029 6.271 0.000

OHYP2 0.035 0.026 1.363 0.173

OCON4 ON

OCON3 0.278 0.033 8.462 0.000

OHYP3 0.023 0.030 0.781 0.435

OCON5 ON

OCON4 0.227 0.036 6.234 0.000

OHYP4 0.079 0.029 2.704 0.007

OCON6 ON

OCON5 0.313 0.037 8.558 0.000

OHYP5 0.085 0.027 3.122 0.002

TCON ON

SEX -0.088 0.025 -3.558 0.000

INCGROUP -0.068 0.022 -3.069 0.002

MH 0.212 0.029 7.363 0.000

THYP ON

SEX -0.232 0.022 -10.397 0.000

INCGROUP -0.024 0.024 -1.027 0.305

MH 0.149 0.025 6.025 0.000

TCON WITH

THYP 0.645 0.023 27.660 0.000

**Kindergarten cohort**

OHYP2 ON

OHYP1 0.228 0.024 9.678 0.000

OCON1 0.031 0.021 1.449 0.147

OHYP3 ON

OHYP2 0.285 0.026 11.168 0.000

OCON2 0.045 0.024 1.853 0.064

OHYP4 ON

OHYP3 0.328 0.026 12.398 0.000

OCON3 0.059 0.025 2.376 0.017

OHYP5 ON

OHYP4 0.383 0.029 13.182 0.000

OCON4 0.039 0.027 1.471 0.141

OHYP6 ON

OHYP5 0.362 0.028 12.811 0.000

OCON5 0.105 0.027 3.854 0.000

OCON2 ON

OCON1 0.210 0.024 8.788 0.000

OHYP1 0.003 0.024 0.111 0.912

OCON3 ON

OCON2 0.202 0.031 6.607 0.000

OHYP2 0.037 0.027 1.358 0.175

OCON4 ON

OCON3 0.245 0.030 8.149 0.000

OHYP3 0.020 0.026 0.784 0.433

OCON5 ON

OCON4 0.255 0.036 7.050 0.000

OHYP4 0.084 0.030 2.798 0.005

OCON6 ON

OCON5 0.287 0.035 8.181 0.000

OHYP5 0.080 0.026 3.071 0.002

TCON ON

SEX -0.122 0.021 -5.912 0.000

INCGROUP -0.038 0.019 -1.963 0.050

MH 0.296 0.025 11.753 0.000

THYP ON

SEX -0.287 0.019 -15.460 0.000

INCGROUP -0.023 0.022 -1.072 0.284

MH 0.224 0.022 10.277 0.000

TCON WITH

THYP 0.670 0.017 39.791 0.000

ON: Regressed on; WITH: Correlation; β: Standardised linear regression coefficient; SEX: Female vs. male; INCGROUP: Income groups; MH: Average of paternal and maternal Kessler 6 scores; OCON: Conduct problems occasion-specific residual at time t; OHYP: Hyperactivity occasion-specific residual at time t; TCON: Random-intercept of conduct problems; THYP: Random-intercept of hyperactivity
